# Metabolic syndrome, endocrine disruptors and prostate cancer associations: biochemical and pathophysiological evidences

**DOI:** 10.18632/oncotarget.16725

**Published:** 2017-03-30

**Authors:** Vincenzo Quagliariello, Sabrina Rossetti, Carla Cavaliere, Rossella Di Palo, Elvira Lamantia, Luigi Castaldo, Flavia Nocerino, Gianluca Ametrano, Francesca Cappuccio, Gabriella Malzone, Micaela Montanari, Daniela Vanacore, Francesco Jacopo Romano, Raffaele Piscitelli, Gelsomina Iovane, Maria Filomena Pepe, Massimiliano Berretta, Carmine D'Aniello, Sisto Perdonà, Paolo Muto, Gerardo Botti, Gennaro Ciliberto, Bianca Maria Veneziani, Francesco De Falco, Piera Maiolino, Michele Caraglia, Maurizio Montella, Rosario Vincenzo Iaffaioli, Gaetano Facchini

**Affiliations:** ^1^ Progetto ONCONET2.0 - Linea progettuale 14 per limplementazione della prevenzione e diagnosi precoce del tumore alla prostata e testicolo, Regione Campania, Italy; ^2^ Division of Medical Oncology, Department of Uro-Gynaecological Oncology, Istituto Nazionale Tumori Fondazione G. Pascale - IRCCS, Naples, Italy; ^3^ Medical Oncology, Abdominal Department, National Cancer Institute G. Pascale Foundation, Napoli, Italy; ^4^ Department of Onco-Ematology Medical Oncology, S.G. Moscati Hospital of Taranto, Taranto, Italy; ^5^ Radiation Oncology, Istituto Nazionale per lo Studio e la Cura dei Tumori Fondazione Giovanni Pascale - IRCCS, Napoli, Italy; ^6^ Pathology Unit, Istituto Nazionale Tumori Fondazione G. Pascale-IRCCS, Naples, Italy; ^7^ Division of Urology, Department of Uro-Gynaecological Oncology, Istituto Nazionale Tumori Fondazione G. Pascale - IRCCS, Naples, Italy; ^8^ Epidemiology Unit, Istituto Nazionale per lo Studio e la Cura dei Tumori Fondazione Giovanni Pascale - IRCCS, Napoli, Italy; ^9^ Psicology Unit, Istituto Nazionale per lo Studio e la Cura dei Tumori Fondazione Giovanni Pascale - IRCCS, Napoli, Italy; ^10^ Department of Molecular Medicine and Medical Biotechnologies, University of Naples Federico II, Naples, Italy; ^11^ Pharmacy Unit, Istituto Nazionale Tumori, Istituto Nazionale Tumori-Fondazione G. Pascale, Naples, Italy; ^12^ Department of Medical Oncology, CRO Aviano, National Cancer Institute, Aviano, Italy; ^13^ Division of Medical Oncology, A.O.R.N. dei COLLI Ospedali Monaldi-Cotugno-CTO, Napoli, Italy; ^14^ Scientific Directorate, Istituto Nazionale per lo Studio e la Cura dei Tumori Fondazione Giovanni Pascale - IRCCS, Napoli, Italy; ^15^ Department of Biochemistry, Biophysics and General Pathology, Second University of Naples, Naples, Italy; ^16^ Association for Multidisciplinary Studies in Oncology and Mediterranean Diet, Piazza Nicola Amore, Naples, Italy

**Keywords:** metabolic syndrome, endocrine disruptors, prostate, cancer, nutrition

## Abstract

This review summarizes the main pathophysiological basis of the relationship between metabolic syndrome, endocrine disruptor exposure and prostate cancer that is the most common cancer among men in industrialized countries. Metabolic syndrome is a cluster of metabolic and hormonal factors having a central role in the initiation and recurrence of many western chronic diseases including hormonal-related cancers and it is considered as the worlds leading health problem in the coming years. Many biological factors correlate metabolic syndrome to prostate cancer and this review is aimed to focus, principally, on growth factors, cytokines, adipokines, central obesity, endocrine abnormalities and exposure to specific endocrine disruptors, a cluster of chemicals, to which we are daily exposed, with a hormone-like structure influencing oncogenes, tumor suppressors and proteins with a key role in metabolism, cell survival and chemo-resistance of prostate cancer cells. Finally, this review will analyze, from a molecular point of view, how specific foods could reduce the relative risk of incidence and recurrence of prostate cancer or inhibit the biological effects of endocrine disruptors on prostate cancer cells. On the basis of these considerations, prostate cancer remains a great health problem in terms of incidence and prevalence and interventional studies based on the treatment of metabolic syndrome in cancer patients, minimizing exposure to endocrine disruptors, could be a key point in the overall management of this disease.

## INTRODUCTION

Prostate cancer represent around 15% of all diagnosed male cancers; in Europe around 2.6 million of new cases, each year, are diagnosed and the last epidemiological studies indicate 35000 new cases in Italy in 2015 [1]. Epidemiologically, the main risk factors for this disease are family history, race and age [2]. Importantly, epidemiological studies focusing on the correlation between geographic position and risk of prostate cancer suggests that western life style could have a central role in the etiology of this disease in fact western men have an incidence rate that is up to 15 times greater than in Asian men and this information suggests that there are still environmental factors or lifestyle, especially nutritional, that may play a key role in prostate cancer phenomenology [3].

### Metabolic syndrome and prostate cancer

The Metabolic Syndrome (MS), also known as insulin resistance syndrome, is a multifactorial disease, that has become quite prevalent within our society, characterized by aggregation of 3 or more metabolic disorders; specifically, these involve the distribution of visceral adipose tissue (waist circumference for woman and men ≥ 88 cm and ≥ 100 cm, respectively), dyslipidemia (based on HDL in woman and man < 50 mg /dl and < 40 mg / dl, respectively with triglycerides > 150 mg/dl for both), intolerance glucose and /or reduced insulin sensitivity (fasting blood glucose > 110 mg/dl), abnormal blood pressure (systolic > 130 mmHg, diastolic > 85 mmHg) [4, 5]. Numerous studies demonstrate that MS predisposing to different chronic disease characteristics of western countries like non-insulin dependent diabetes (NIDS) [6], psoriasis [7], obesity [8], cardiovascular diseases (CVD) [9, 10], osteoarthritis [11], neurodegenerative diseases like Alzheimer syndrome [12], the most common hormone-dependent tumours like breast [13] and prostate [14], as well as pancreas [15], colorectal cancer [16] and hepatocellular carcinoma [17]

**Figure 1:**
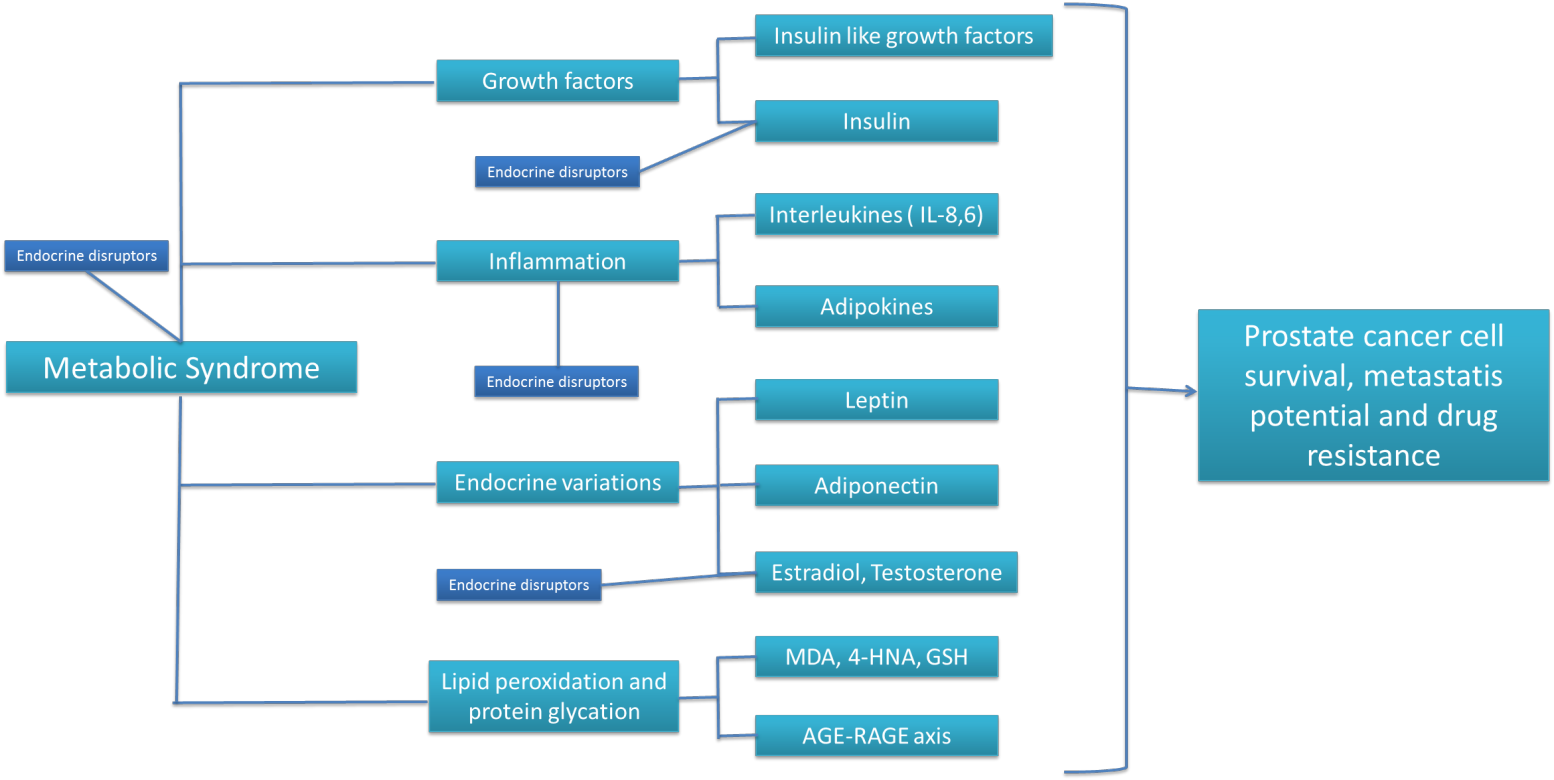
Putative relations among metabolic syndrome, endocrine disruptors, growth factors, inflammation, lipid/protein chemical modifications and prostate cancer biology

Recently studies showed a convincing association between MS and cancer risk, including prostate [18]. Among clinical characteristics of MS, insulin resistance with consequent high insulin levels in the blood represents one of the most important pathogenetic mechanism of MS; in fact, insulin stimulate lipogenesis, steroidogenesis, protein synthesis and, as growth factor molecule, it stimulates cellular proliferation with anti-apoptotic activities especially in hormone-independent prostate cancer cells [19]. Among factors triggering MS, also prostate cancer treatments could influence its incidence in these patients in fact more than 50% of prostate cancer patients following Androgen Deprivation Therapy (ADT) for more than 6 months develop MS, increases body mass index (BMI), triglycerides and total cholesterol levels, increase LDL/HDL ratio and insulin levels (insulin resistance) with central influence to the all-cause morbidity and mortality in these patients. From a clinical point of view MS in an important prognostic factor in prostate cancer in relation to its close link with cardiovascular diseases (CVDs), castration-resistant prostate cancer (CRPC) [20], PSA recurrence and metastases [21]. Biologically, how MS factors could influence prostate cancer cell survival, metastasis and drug resistance? As shown in Figure 1, MS has different connection points to prostate cancer biology with affections on growth factors, adipokines, interleukines, sexual hormones, endocrine disruptors, lipids and proteins modified by pro-oxidative microenvironment (as example peroxidized lipids and glycated proteins). Another recent epidemiological study demonstrate that African Americans affected by MS, that specifically have abdominal obesity and hypertension, has a 90% increased risk of prostate cancer development [22]. Moreover, men with MS has prostate cancer with more advanced Gleason score with a greater risk both of cancer progression after radical prostatectomy that of prostate cancer-related death [23]. Regarding the axis inflammation - prostate cancer, it is well known that chronic inflammation to prostate gland, that is common in patients affected by MS, is associated with a higher concentration of cytokines, interleukines and growth factors that stimulate prostate cell division with increased likelihood of accumulating point mutations and epigenetic modification like DNA hypermethylation of caretaker genes *GSTP1*, *RASSF1A*, and *APC*.

### Growth factors and prostate cancer

Several studies demonstrated that progression of prostate cancer is closely related to plasma growth factors [24]. Insulin is one of the most important and studied growth factor correlated to prostate cancer as well as MS, in fact, men with high serum insulin levels, as well as abdominal obesity associated with high waist-to-hip ratio (WHR) have a greater risk of prostate cancer incidence [25]. Moreover, a growth factor structurally similar to insulin peptide, called Insulin Like Growth factor type 1 (IGF-1), is inversely associated with all-cause mortality in men affected by prostate cancer [26] if fact it is a candidate prognostic marker in patients affected by advanced disease. Another recent study associate C-peptide plasmatic levels (as indirect marker of insulin) with higher risk of death in prostate cancer patients [27]. Biologically, these correlations are related to the mitogen activity of these molecules on cancer cells because, through one transmembrane-segment receptors (1STMR), they stimulate mTOR activation that is a common therapeutic target of several cancer the prostate one [28].

### Inflammation and prostate cancer

As recently well demonstrated, chronic inflammation contributes to prostate cancer initiation and progression [29]. For example, studies of genetic susceptibility have shown that subjects with specific mutations in genes coding to protein with a key role in the inflammation network like IL-6, MSR1, TNFα, and IL-8 have greater risk of incidence of this cancer compared to wild type patients [30] indicating an essential role of the inflammation in the malignant transformation of prostate cells. From a biochemical point of view, inflammation status is linked to visceral obesity with the presence of a pro-inflammatory microenvironment in fact abdominal obesity, especially in subjects affected by MS, is associated with a state of chronic inflammation based on the accumulation of immune cells (as example macrophages and leucocytes) among adipocytes; these cells produce several pro-inflammatory cytokines and interleukines like IL-6, IL-8, TNFα and CRP (C-reactive protein) [31] This mechanism, then, leads to the formation of fat cells surrounded by macrophages, called “crown adipocytes”, that influence in autocrine and paracrine manner the peri-adipocyte microenvironment with consequent inhibition of adiponectin gene expression. Adiponectin is one of the most important activator of AMPK and PPARα stimulating fatty acid oxidation, reduction of inflammation and regulation of cancer survival [32].

Biological evidences that correlate inflammation with prostate cancer initiation, progression and prognosis are based on different cytokines like IL-6, IL-8, MIC-1. IL-6 is a multifunctional cytokine produced by macrophages, endothelial cells, T lymphocytes but also cancer cells like breast and prostate [33]. In fact IL-6 can be produced by both androgen sensitive than the insensitive prostate cancer cells, for example under TLR4 activation from LPS binding and up-regulation of both ligand and its receptor are overexpressed in prostate cancer cells (called, as example, LNCaP and DU145 cell lines) but also in a disease that precede the development of prostate adenocarcinoma called high-grade prostatic intraepithelial neoplasia (PIN). Moreover, IL-6 could have a crucial role in the activation of androgen receptor (AR) in cancer cells [34]. Clinically, patients with hormone-refractory disease and metastatic prostate cancer has high serum levels of IL-6 in fact, interestingly, it stimulates a crucial biological process in cancer called epithelial-mesenchymal transition (EMT) as well as bone metastasis promoting localization of metastatic prostate cancer cells in the bone in fact IL-6 has been proposed as a new marker of morbidity in prostate cancer men [35]. Moreover, high IL-6 serum levels were observed in patients with a poor responsiveness to chemotherapy and affected by castration-resistant prostate cancer (CRPC) ; in fact it protects androgen sensitive cells (called LNCaP) from apoptosis induced by androgen deprivation and these effects are principally mediated by Stat3 activation [36]. In last years, it was observed that IL-6 play a crucial role in MDR (multi drug resistance) processes; in fact stimulation of androgen sensitive and insensitive prostate cancer cells with IL-6 determine, trough nuclear factor kappaB (NF-kappaB) activation, induce resistance to docetaxel treatments [37]. NF-kappaB is a molecular complex whose nuclear localization is associated with prostate cancer [38]; specifically, overexpression of a NF-kappaB subunit, called RelB, is well associated with Gleason scores in prostate cancer patients indicating a key role of this inflammation marker in the progression of the disease. Among cytokines network involved in prostate cancer, a cytokine produced by macrophages called Macrophage inhibitory cytokine type 1 (MIC-1) is one of the most studied protein in the progression of this disease; gene overexpression of MIC-1 is associated with prostate cancer tissue and with Gleason score (> 5 G.S) indicating its possible role as clinical marker in diagnosis and prognosis of prostate cancer [39]. IL-8 is a well known pro-inflammatory chemokine with key roles in the activation of intracellular signaling pathways involved in the activation of receptors called CXCR1 and CXCR2. Overexpression of IL-8 as well as its receptor has observed clinically in prostate cancer tissue and tumour associated macrophages indicating its role in tumour microenvironment formation [40] and its biochemical functions are recently associated in the formation of a tumor biology characterized by a resistance to androgens levels [41]. Patients with MS has generally high levels of glucose in the blood and a strong oxidative stress mainly at level of membrane phospholipids [42]. One of the most important pro-inflammatory stimuli in prostate cancer is due to Advanced Glycation End Product (AGE) and lipid peroxides like Malondialdehyde (MDA) and 4-hydroxynonenal (4-HNA). AGEare constantly generated under a high level of glucose concentration (hyperglycemia) *via* nonenzymatic pathway; this reaction is called glycation. During glycation process, glucose can bind to proteins modifying their functions. Interestingly, MS patients has levels of AGE, MDA and 4-HNA higher compared to subjects without MS [43]. These molecules principally through NFĸB activation are pro-inflammatory molecules in prostate cells in fact it has recently shown that receptor of AGE (RAGE) is overexpressed in prostate cancer cells so it could have a central role in the initiation as well as progression of prostate cancer [44]. Moreover, recently, it was seen that higher levels of plasma carboxymethyllysine (CML) (a major end-stage AGE) were associated with increased risk of prostate [45]. Also products of lipid peroxidation are related to prostate cancer; in fact plasma MDA levels are higher in prostate cancer patients compared to non-cancer patients [46] and more clinical studies could be useful to associate the lipid peroxidation products with preventive and predictive markers of prostate cancer in male affected by MS.

### Endocrinology of obesity and prostate cancer

Prostate cancer patients affected by MS has high levels of leptin and low levels of adiponectin [47]. Clinically, greater serum levels of leptin and lower levels of adiponectin are associated with prostate cancer initiation and progression. Leptin, an hormone activating the same pathway of insulin and IGF-1, stimulate survival of prostate cells hormone independent and increased blood levels of leptin were observed in patients with an aggressive biology of prostate cancer [48]. Recently it was demonstrated a crosstalk among leptin and insulin and/or IGF-1 in cancer cells due to the same stimulation of PKB-mTOR pathway. High levels of leptin in patients with MS may be due to a phenomena called leptin resistance that can be related to the high fructose intake that reduce the responsiveness of the leptin receptor at the level of the blood brain barrier (BBB) altering the natural feedback mechanisms to control its blood levels [49]. In contrast, adiponectin has lower concentrations in MS patients [50]. These facts could be related to regulation of gene expression because insulin and IGF-1, principally trough IRS-1-PKB -FoX01 pathway, growth factors trough Ras-ERK-NFATc4 pathway, IL6 and IL-8 principally by STAT3-cFOS-FoX01 axis and free fatty acids trough pathway mediated by PKA-CREB-ATF3 determine a significant inhibition of adiponectin gene expression. Adiponectin is essential in prostate cancer prevention and management because it has anti angiogenic activity also trough activation of AMPK-TSC pathway (resulting in inhibition of mTOR activation) in fact patients affected by aggressive and metastatic prostate cancer has lower blood levels of adiponectin.

### Endocrine disruptors and prostate cancer

**Figure 2:**
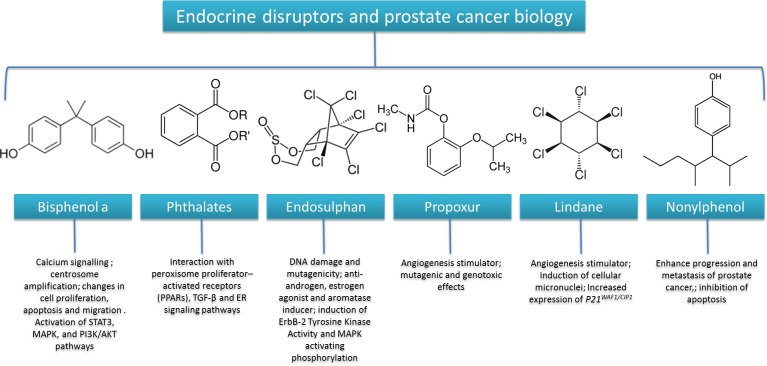
Relationships between endocrine disruptors and prostate cancer biology

Endocrine disruptors are exogenous substances (i.e drugs, pesticides, plastic additives, organic pollutants but also natural plant substances) that interfere with the synthesis, secretion, transport, binding to receptors, action or elimination of natural human hormones; most of these are xenoestrogens or antiandrogens. Essentially, we are daily exposed to very low concentration of multiple endocrine disruptors and the most known and studied are available and released from the following sources: Perfumes (may contain phthalates), pesticides, insecticides (may contain endosulphan) sunscreen (may contain parabens, Nonylphenol), Drugs (may contain Nonylphenol), Fungicides, epoxy resins, flame retardants (PCBs, PBBs), Recycled Paper, Plastic bottles (polycarbonate, bisphenol) lubricants (may contain Nonylphenol), plastics and food packaging, hospital instruments (may contain phthalates) [51]. Recent biological and epidemiological studies correlate endocrine disruptors exposure with obesity, MS, type 2 diabetes and cancer [52]. As example, bisphenol A (Figure 2), as additive for polycarbonate plastics (bottles, food containers, interior coatings of cans), is a common endocrine disruptor correlated with several cancers [53]. Laboratory animals exposed to low doses of bisphenol a develop diabetes, reproductive problems (precocious puberty, reduced sperm shame), obesity, breast and prostate cancer however human studies that correlate bisphenol a exposure and cancer risk are scarce. Biologically, it is well known that bisphenol A, at very low doses, induces prostate cancer cells migration modulating the ion channel protein expression involved in cancer cell migration [54]. Moreover, just one study observed that higher levels of urinary bisphenol a are shown in prostate cancer patients compared to non-cancer patients indicating this endocrine disruptor as an independent prognostic marker in prostate cancer but more clinical studies are needed [55].

Other common endocrine disruptors are phthalates (Figure 2), molecules with demonstrated estrogenic effects both in breast and prostate cancer cells also with possible implication in the etiology of hormone-independent cancer [56]. Generally, phthalates impair testicular function and have been associated with anti-androgenic effects in humans and recently, in the very few human studies conducted, they were correlated with abdominal obesity and insulin resistance due to their interaction with peroxisome proliferator-activated receptors (PPARs) that regulate lipid and glucose metabolism [57] but more clinical studies about phthalates exposure and MS are needed. As estrogen-like substances, phthalates can induce growth of human hormone dependent prostate cancer cells by acting on the crosstalk between TGF-β and ER signaling pathways [58]. Among common endocrine disruptors, endosulfan (Figure 2) is one of the most studied in oncology; endosulfan is an organochlorine insecticide and acaricide with similar estrogenic effects [59] and maternal exposure to this molecule cause cryptorchidism which is well-known risk factor for testicular cancer [60]. Endosulfan exposure was recently associated to higher risk of prostate cancer incidence in humans probably due to its mutagenic [61], anti-androgen, estrogen agonist and aromatase inducer activites [62]; endosulfan increase activating phosphorylation of EGFR type 2 and it increases activity of several oncogenes like MAPK in prostate cancer cells [63]. Endosulfan with other xenoestrogens like lindane and propoxur, act as angiogenesis stimulator both *in vitro* as well as *in vivo* models but more molecular and biochemical studies are needed [64]. Another industrial chemical with estrogenic activity is nonylphenol (Figure 2); considering its high hydrophobicity, nonylphenol accumulate in several matrices like soils, rivers, as well as, food and drinking water and it was found in human plasma, breast milk [65]. Due to its molecular conformation similar to estradiol, nonylphenol exhibits several prostate cellular effects; in fact it stimulates cell survival and metastasis of hormone-dependent prostate cancer cells line by reducing mRNA levels of tumour suppressor genes like p27 and Bax [66] and all of these effects are similar to those observed for dihydrotestosterone (DHT) indicating the strong hormonal impact of this industrial chemical in prostate cancer biology. Recently, it was demonstrated a synergistic pro estrogenic activities of nonylphenol and bisphenol a co-incubated with human prostate cancer cells indicating interesting ideas for research about interactions between different endocrine disruptors [67]. However, on the basis of these findings, more phenomenological studies are required to understand the multiple effects of endocrine disruptors on metabolic risk factors for cancer like MS, insulin resistance, hormonal changes and on relative risk of incidence and tumor recurrence, understanding if multiple exposure to different endocrine disruptors could have synergistic carcinogenic or pro angiogenesis effects in humans. Interestingly, it would require intervention studies to understand if some natural molecules can counteract the hormonal and pro oncogenic effects of endocrine disruptors as recently seen for curcumin, a bioactive anti-inflammatory compound that has shown interesting protective properties against cancer proliferative effects of bisphenol a trough its modulation of miR-19/PTEN/AKT/p53 axis [68].

### Nutrition and prostate cancer

Despite the large amount of data related to biochemical and metabolic pathophysiology of prostate cancer and the relationships between environmental factors and prostate somatic mutations, there are very few studies focusing on the effects of diet on prostate cancer prognosis. However, several studies shown that incidence of prostate cancer especially one with a more aggressive tumor biology is higher in regions where man eating in “western manner” compared to a more vegetarian diet style typical of oriental countries [69]. The World Cancer Research Founding (WCRF) in 2007 describe clearly that foods higher in lycopene and selenium levels has probably protect against prostate cancer incidence instead foods higher in calcium could be involved in the progression of this cancer. Moreover, several epidemiological studies has shown protective effects of tocopherols (vitamin E), pulses, soy foods in prostate cancer incidence [70]. Considering few studies demonstrating protective role of a traditional Mediterranean diet style against prostate cancer initiation it can be assessed, from a nutritional point of view, that the intake of tocopherols and cooked tomatoes is associated to a lower prostate cancer risk and the intake of dairy products is associated to a greater prostate cancer risk [71]. Interestingly, changing from a Western to a Mediterranean diet can reduce the incidence of prostate cancer by around 10% [72] so it could be important to consider eventual changes of dietary patterns in prostate cancer prevention. However, considering this epidemiological considerations, molecular determinants in foods that change biology of prostate tissue and prostate cancer as can be summarized: fatty acids (EPA, DHA, arachidonic acid, ALA), Vitamin D, selenium, lycopene, calcium, brown rice. Relatively to fatty acids, as well known, their biological effects related also to prostate cancer and MS depending from their chemical nature but MS patients has a lipidomic profile based on low levels of glycerolipids and greater plasma levels of glycerophospholipids and saturated fatty acids compared to health patients [73]. However, EPA and DHA, two common products of long chain n-3 fatty acids metabolism, protects against prostate cancer and MS due to their anti-inflammatory effects based also in the inhibition of COX-2 and PPARc [74]. Recently, it was demonstrated that n−3 PUFAs protect against MS due to their anti-inflammatory, platelet activating properties that improve endothelial function and regulate blood pressure also through inhibition of lipogenesis and activation of lipid oxidation [75]. However, it is important to assess that not all clinical studies are consistent about protective effects of omega-3 fatty acids, in fact, high intake of omega-3 fats correlate to increased prostate cancer risk indicate that very high doses of these fatty acids could be involved in prostate tumorigenesis so recommendations to increase omega-3 intake, specially from supplements, should be taken with caution since these indications. [76].

What about arachidonic acid? It is a saturated fatty acid, central promoter of the series 6 prostaglandins synthesis in cells. A common metabolite of arachidonic acid, derived from lipoxygenases activities, called 12-hydroxy eicosatetraenoic acid (12-HETE) is well correlated to prostate cancer initiation and progression, in fact higher tissue levels of 12-HETE were correlated with prostate cancer [77]. On the basis of this information it could be assessed that lipoxygenases, specifically the primary metabolite from arachidonic acid like 12-HETE could have a critical role in prostate cancer progression [77]. Relatively to Vitamin D, it is well known that low levels of this molecule are associated with a favorable risk of prostate cancer risk even if not in all segments of the population [70]. Interestingly, Vitamin D3 inhibit prostate cancer survival and metastasis in several cellular and preclinical studies [78]; moreover it inhibits prostate cancer cell proliferation, induces differentiation and apoptosis and this molecular mechanisms explain in same parts why low levels of plasmatic vitamin D is significantly associated to increase prostate cancer risk and a more aggressive biology of cancer [79, 80]

Apart this associations, as described in WCRF and European Code Against Cancer recently, food supplements should be used with extreme caution for prevention or management of cancer; as example, oncologists correlate assumption of supplements of selenium and vitamin E with an higher risk of prostate cancer [81]. So on the basis of these considerations selenium supplementation could not be useful for cancer prevention in fact man with greater risk of prostate cancer with an aggressive biology have high serum selenium levels. However, it is important to specify that selenium, as well as vitamin E, taken naturally from foods and not from supplements, has an opposite function on prostate cancer prognosis. In fact the WCRF dictates reported for protection from prostate cancer is important to consume frequently foods contains naturally high selenium content [82] so, from a nutritional point of view, it could be useful to consume up to 3 tablespoons (daily) of extra virgin olive oil (possible the cold pressed one) or a handful of sesame seeds or pumpkin, walnuts and almonds which are naturally rich in selenium and vitamin E. About the relationship between calcium, dairy products consume and risk of prostate cancer, researchers reported, in summary, that the frequent intake of these types of foods may increase the risk of incidence of this cancer [82];The quantity of milk consume is so important, in fact it was shown that men who drank daily more than 2 glasses of milk has an higher risk of incidence of an aggressive prostate cancer compared to patients that consume a lesser amount [83] and the reasons about this association could be related to high calcium levels and prostate cancer metabolism; in fact a recent research demonstrate that man taking around 2 grams daily of calcium has an higher risk of prostate cancer incidence compared to man who consume a quarter of the quantity, corresponding to around 0,5 grams daily [84]. Another essential nutrient factor related to prostate cancer is lycopene, a strong antioxidant carotenoid that accumulates in various tissues of the body including testicular, breast, liver, kidneys and prostate; the main sources of lycopene are tomato sauce, red tomatoes especially, melons, pink grapefruit, guava, red carrots, papaya, strawberries and cherries. However, the biological role of lycopene in prostate cancer has however a series of unclear points [85] but recent studies demonstrate that man taking lycopene naturally from a proper diet have lower risk to develop lethal prostate cancer and metastasis. Moreover, a recent meta-analysis has shown that around 10-20 % lower risk of prostate cancer incidence is seen in man taking frequently tomato in fact man with higher blood levels of lycopene have a significant lower risk of developing this cancer [86].

Finally, recent research associate dietary rice bran intake with protective effect against several diseases like prostate, breast and colon cancer. Its chemo preventive potential is biologically related to several polyphenol, flavonoids and fatty acid (as example tricin, ferulic acid, oryzanol, tocotrienols etc etc…). Several studies indicate that these components of brown rice inhibit cancer cell proliferation, survival and angiogenesis [87]. Relatively to prostate cancer, it is interesting that polyphenols and flavonoids derived from brown rice, primary due to the activation of pathways responsive to energy deprivation (specifically based on the activation of Phospho-AMP-activated kinase α), reduce prostate cancer cell growth in time and concentration dependent manner [88, 89].

## CONCLUSIONS

Based on the high incidence of central obesity and MS in the world with special reference to western countries, and considering the well-evident connection between these factors and prostate cancer cell metabolism, it could be crucial to understand the connections between single MS determinants and prostate cancer biology and how to limit environmental exposure to endocrine disruptors that have a key role in the phenomenology of this cancer. Moreover, on the basis of the data shown in this review, it could be useful to design new human prospective studies for understand how single or multiple exposure to endocrine disruptors could change relative risk of prostate cancer. It would be extremely necessary, therefore, to initiate nutritional intervention studies to fight MS in these patients, specifically by changing from a Western to a Mediterranean style diet that follow the general recommendations of WCRF of 2007 (limit the high caloric density foods, eliminate preserved meat, limit red meat, avoid the consumption of sugary drinks), prefer vegetal foods, with particular reference to those rich in tocopherol, lycopene and anti-inflammatory flavonoids like those coming from the chaff of whole grains as example brown rice, ancient grains, millet, quinoa, amaranth and buckwheat. Moreover, it should be required a drastic reduction in exposure to certain endocrine disruptors as example mainly through drastic changes in lifestyles that must initiate from gestational life (as example, reduction of plastic use thus favoring glass containers ; reduction to flame retardant exposure because endocrine disruptors that are content could accumulate in household dust and spread in the house environment so it could be important always to choose flame-retardant-free objects etc..). Lifestyle changes could reduce the bio-accumulation of these molecules in the organs reducing the relative risk of developing hormone-sensitive tumors in adulthood or anyway getting a better overall management in prostate cancer patients.
