# Correction: Metabolic syndrome, endocrine disruptors and prostate cancer associations: biochemical and pathophysiological evidences

**DOI:** 10.18632/oncotarget.20631

**Published:** 2017-09-05

**Authors:** Vincenzo Quagliariello, Sabrina Rossetti, Carla Cavaliere, Rossella Di Palo, Elvira Lamantia, Luigi Castaldo, Flavia Nocerino, Gianluca Ametrano, Francesca Cappuccio, Gabriella Malzone, Micaela Montanari, Daniela Vanacore, Francesco Jacopo Romano, Raffaele Piscitelli, Gelsomina Iovane, Maria Filomena Pepe, Massimiliano Berretta, Carmine D’Aniello, Sisto Perdonà, Paolo Muto, Gerardo Botti, Gennaro Ciliberto, Bianca Maria Veneziani, Francesco De Falco, Piera Maiolino, Michele Caraglia, Maurizio Montella, Rosario Vincenzo Iaffaioli, Gaetano Facchini

**Affiliations:** ^1^ Progetto ONCONET2.0 - Linea progettuale 14 per l’implementazione della prevenzione e diagnosi precoce del tumore alla prostata e testicolo, Regione Campania, Italy; ^2^ Division of Medical Oncology, Department of Uro-Gynaecological Oncology , Istituto Nazionale Tumori ‘Fondazione G. Pascale’ - IRCCS, Naples, Italy; ^3^ Medical Oncology, Abdominal Department, National Cancer Institute G. Pascale Foundation, Napoli, Italy; ^4^ Department of Onco-Ematology Medical Oncology, S.G. Moscati Hospital of Taranto, Taranto, Italy; ^5^ Radiation Oncology, Istituto Nazionale per lo Studio e la Cura dei Tumori ‘Fondazione Giovanni Pascale’ - IRCCS, Napoli, Italy; ^6^ Pathology Unit, Istituto Nazionale Tumori “Fondazione G. Pascale”-IRCCS, Naples, Italy; ^7^ Division of Urology, Department of Uro-Gynaecological Oncology , Istituto Nazionale Tumori ‘Fondazione G. Pascale’ - IRCCS, Naples, Italy; ^8^ Epidemiology Unit, Istituto Nazionale per lo Studio e la Cura dei Tumori ‘Fondazione Giovanni Pascale’ - IRCCS, Napoli, Italy; ^9^ Psicology Unit, Istituto Nazionale per lo Studio e la Cura dei Tumori ‘Fondazione Giovanni Pascale’ - IRCCS, Napoli, Italy; ^10^ Department of Molecular Medicine and Medical Biotechnologies, University of Naples “Federico II”, Naples, Italy; ^11^ Pharmacy Unit, Istituto Nazionale Tumori, Istituto Nazionale Tumori-Fondazione G. Pascale, Naples, Italy; ^12^ Department of Medical Oncology, CRO Aviano, National Cancer Institute, Aviano, Italy; ^13^ Division of Medical Oncology, A.O.R.N. dei COLLI “Ospedali Monaldi-Cotugno-CTO”, Napoli, Italy; ^14^ Scientific Directorate, Istituto Nazionale per lo Studio e la Cura dei Tumori ‘Fondazione Giovanni Pascale’ - IRCCS, Napoli, Italy; ^15^ Department of Biochemistry, Biophysics and General Pathology, Second University of Naples, Naples, Italy; ^16^ Association for Multidisciplinary Studies in Oncology and Mediterranean Diet, Piazza Nicola Amore, Naples, Italy; ^17^ Scientific Directorate, Istituto Nazionale per lo Studio e la Cura dei Tumori ‘Regina Elena’ - IRCCS, Roma, Italy

**This article has been corrected:** The institutional affiliation information for Dr. Gennaro Ciliberto was updated to include affiliation #17.

The authors sincerely apologize for this oversight.

Original article: Oncotarget. 2017; 8:30606-30616. https://doi.org/10.18632/oncotarget.16725

